# Supplementary figures and images for: Development of a Clinical MALDI-ToF Mass Spectrometry Assay for SARS-CoV-2: Rational Design and Multi-Disciplinary Team Work
Source: Diagnostics (Basel). 2020 Sep 24;10(10):746. doi: 10.3390/diagnostics10100746 (PMC7600155; doi:10.3390/diagnostics10100746)

A

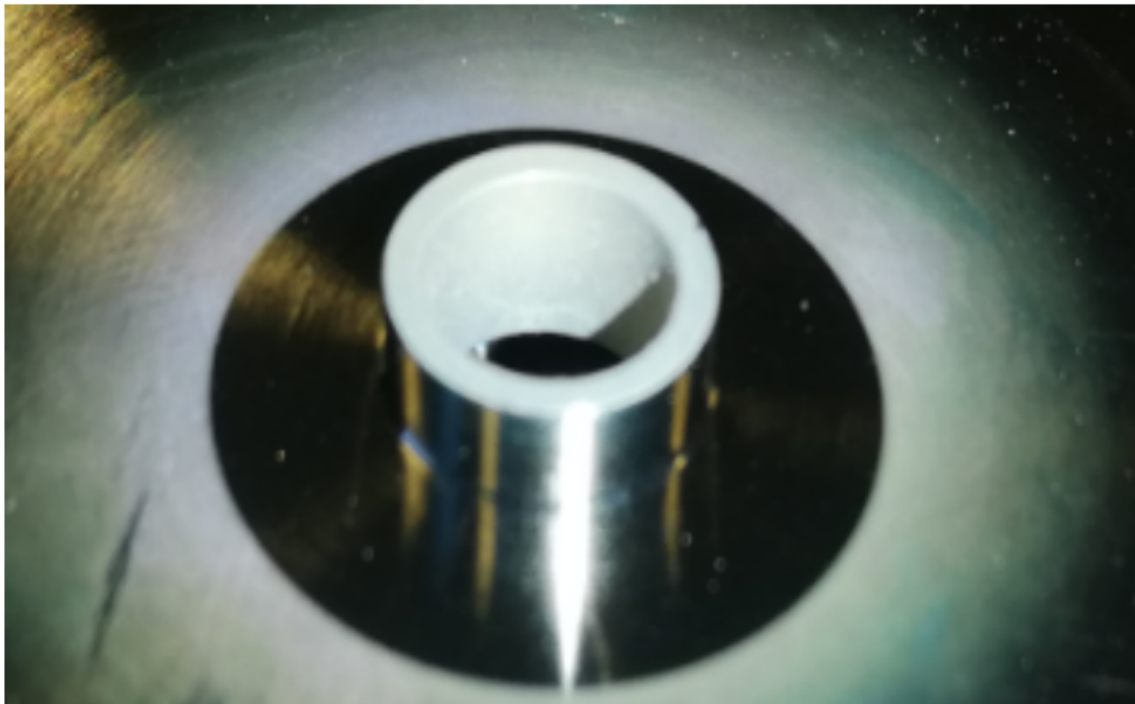

B

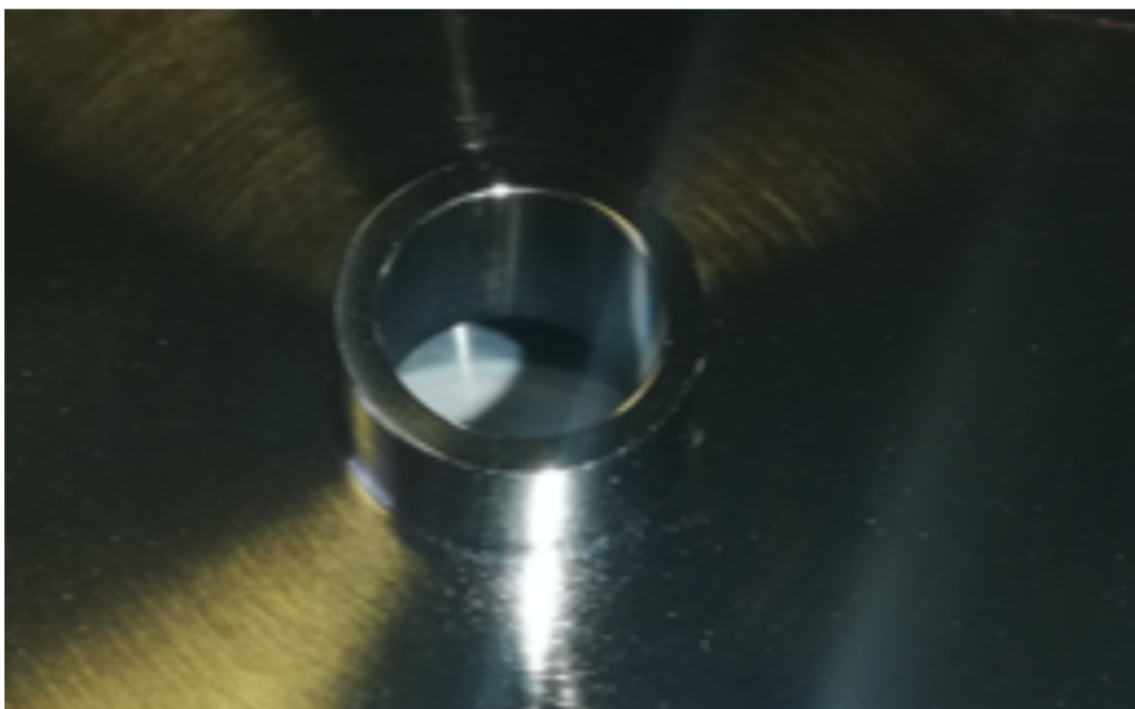

**Figure S1.** Fouling of primary lens from high throughput analysis of samples.

Supplement: Supplementary file 1 [file diagnostics-10-00746-s001.pdf]
